# Supplementary material for: FastqPuri: high-performance preprocessing of RNA-seq data
Source: BMC Bioinformatics. 2019 May 3;20:226. doi: 10.1186/s12859-019-2799-0 (PMC6500068; doi:10.1186/s12859-019-2799-0)
Supplement: Supplementary file 2 — Archive of FastqPuri. Archive containing all files needed to install and run FastqPuri v1.0.6. Date stamp March 22, 2019. (GZ 47,819 kb) [file 12859_2019_2799_MOESM2_ESM.gz › FastqPuri-1.0.6/latex/trim_8c__incl.pdf]

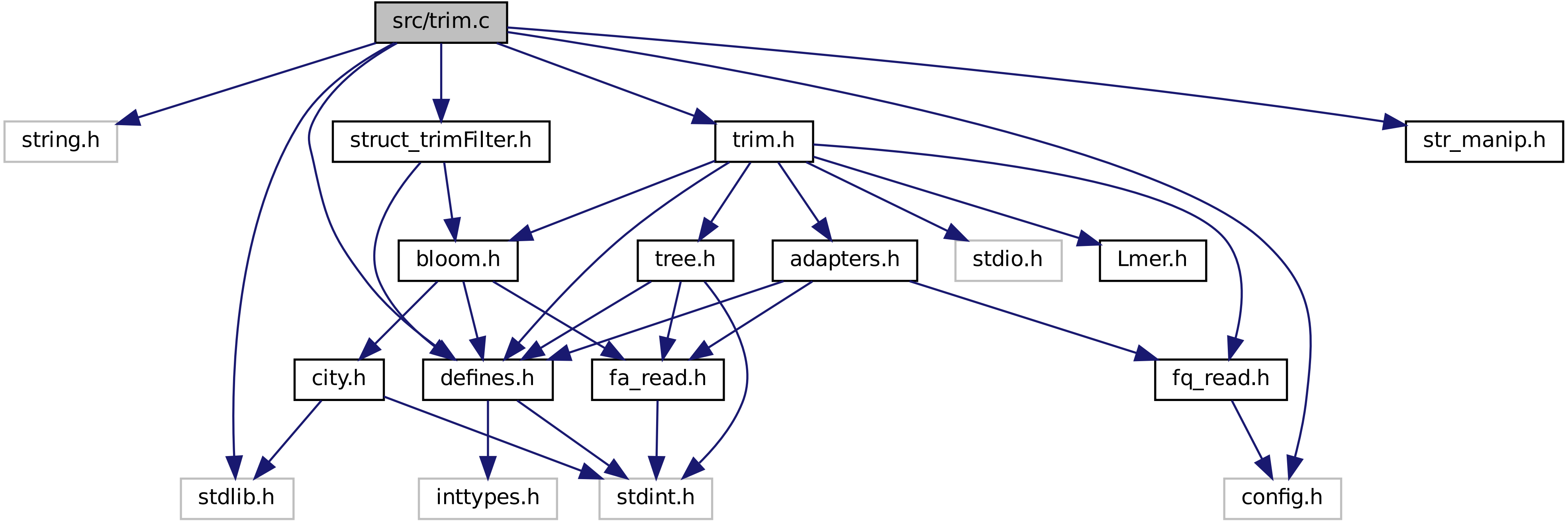
src/trim.c
string.h
struct_trimFilter.h
trim.h
str_manip.h
bloom.h
tree.h
adapters.h
stdio.h
Lmer.h
city.h
defines.h
fa_read.h
fq_read.h
stdlib.h
inttypes.h
stdint.h
config.h
